# Steaming and Sodium Bicarbonate-Assisted Aqueous Extraction of *Panax notoginseng* Stems and Leaves: Evaluation of Xanthine Oxidase Inhibitory Potential

**DOI:** 10.3390/foods15152623

**Published:** 2026-07-27

**Authors:** Zuoming Cao, Yan Wang, Zuoting Yang, Xiaoyu Gao, Jun Sheng, Yang Tian, Lei Peng

**Affiliations:** 1 Key Laboratory of Development and Utilization of Food and Medicinal Resources, Ministry of Education, Yunnan Agricultural University, Kunming 650201, China; 15298198413@163.com (Z.C.); 2018039@ynau.edu.cn (Y.W.); yzt94448@163.com (Z.Y.); 2018014@ynau.edu.cn (X.G.); shengjun_ynau@163.com (J.S.); 2 Engineering Research Center of Development and Utilization of Food and Drug Homologous Resources, Ministry of Education, Yunnan Agricultural University, Kunming 650201, China; 3 Yunnan Key Laboratory of Precision Nutrition and Personalized Food Manufacturing, Yunnan Agricultural University, Kunming 650201, China; 4 College of Food Science and Technology, Yunnan Agricultural University, Kunming 650201, China

**Keywords:** *Panax notoginseng* stems and leaves, hyperuricemia, inhibition kinetics, metabolomics, network pharmacology, molecular docking

## Abstract

*Panax notoginseng* stems and leaves are abundant agricultural residues generated during root harvest, rich in flavonoids yet remain largely underutilized as a valuable phytochemical source. This study optimized a green steaming-sodium bicarbonate aqueous extraction process via orthogonal design. Optimal conditions (steaming 1 h, 1.0% food-grade NaHCO_3_, boiling extraction 2 h, 1:20 g/mL) achieved a solid yield of 13.50 ± 0.41%, significantly higher than conventional water extraction (7.98 ± 0.41%, *p* < 0.05), demonstrating enhanced recovery of bioactive compounds. The extract exhibited 91.95 ± 0.08% xanthine oxidase inhibition (IC_50_ = 5.0 mg/mL) with reversible mixed-type inhibition (*K*_i_ = 4.99 mg/mL). UHPLC-MS/MS identified 3392 metabolites, predominantly flavonoids, alkaloids, and organic acids with potential XO inhibitory activity. Network pharmacology and molecular docking revealed multi-target interactions involving ADRA2A, ADA, GAA, PNP, and PTGS2 enriched in purine metabolism and AGE-RAGE signaling pathways, with quercetin 3-Gentiobioside exhibiting the strongest binding affinity and favorable ligand-target interactions (ΔG = −12.91 kcal/mol). This eco-friendly strategy employs only pure water and food-grade additives, efficiently concentrating xanthine oxidase inhibitors and offering a clean-label, sustainable approach for high-value utilization of *P. notoginseng* agricultural residues. These findings provide a pharmacological basis for developing functional foods targeting hyperuricemia and associated metabolic disorders.

## 1. Introduction

*Panax notoginseng* belongs to classic medicinal and edible plants widely cultivated across China. The underground root tuber has long been the primary medicinal raw material, whereas aboveground stems and foliage are mostly discarded as agricultural waste after harvest [1]. Published research confirms that aerial tissues accumulate flavonoids at concentrations two to three times greater than root tissues; these flavonoid compounds, together with ginsenosides, exhibit strong antioxidant capacity and effective inhibitory effects against multiple enzymes [2,3]. In addition to flavonoids and ginsenosides, *P. notoginseng* also contains polysaccharides, amino acids, and trace elements, which collectively contribute to its diverse pharmacological properties, including anti-inflammatory, cardio-cerebrovascular protective, and immunomodulatory activities [4,5]. Even so, few research efforts have systematically optimized extraction parameters for this stem and leaf waste, resulting in poor utilization efficiency of these byproducts. Conventional extraction of bioactive components from *P. notoginseng* primarily relies on organic solvents (e.g., ethanol, methanol), heat-reflux, or maceration, which suffer from drawbacks such as high solvent consumption, environmental concerns, and potential solvent residues [6,7]. Establishing high-efficiency, environmentally friendly extraction strategies is therefore essential to elevate the economic value of *P. notoginseng* agricultural residues. To address this, we developed a green steaming-food-grade sodium bicarbonate synergistic aqueous extraction method, which eliminates organic solvents, utilizes GRAS (Generally Recognized as Safe) reagents, and integrates steam pre-treatment to enhance cell wall disruption and flavonoid release, representing a distinct advance over conventional solvent-based and unassisted aqueous approaches [8].

Hyperuricemia (HUA) has become one of the most prevalent metabolic disorders worldwide, with its global burden rising substantially over the past decades [9]. Xanthine oxidase (XO) functions as the key rate-limiting enzyme that catalyzes uric acid formation, which makes it a vital therapeutic target for developing dietary products with uric acid-lowering bioactivity [10,11,12]. First-line clinical agents allopurinol and febuxostat carry side effects such as hepatic and renal toxicity; thus, exploring safe, long-term edible natural XO inhibitors has attracted extensive research attention [13,14,15]. By-products of medicinal-edible plants are safe food-derived bioactive resources for natural enzyme inhibitor exploitation [16,17]. Although previous work verified that combined steaming-alkali-assisted aqueous extraction markedly improves flavonoid yield from homologous raw materials [18,19,20], no study has yet optimized this green process specifically for *P. notoginseng* stems and leaves or evaluated its XO-inhibitory activity. This gap is significant because the unique phytochemical profile of *P. notoginseng* stems and leaves-distinct from roots in both composition and abundance, warrants dedicated methodological optimization to maximize bioactive recovery while maintaining food-grade safety.

Moreover, the hypouricemic material basis and multi-target mechanism of *P. notoginseng* stems and leaves remain unclear, hindering their high-value utilization. Therefore, we hypothesized that the optimized steaming-sodium bicarbonate-assisted aqueous extract of *P. notoginseng* stems and leaves would exhibit potent XO inhibitory activity in vitro, and that the underlying hypouricemic effects could be preliminarily predicted through metabolite profiling and in silico multi-target analysis. Herein, XO inhibitory activity was set as the key indicator to optimize the steaming-sodium bicarbonate-assisted aqueous extraction procedure. The specific aims of this study were: (i) to optimize the extraction parameters using single-factor and orthogonal designs; (ii) to characterize the inhibition kinetics of the resulting extract; (iii) to identify the bioactive metabolome via UHPLC-MS/MS widely targeted metabolomics; and (iv) to elucidate the potential hypouricemic mechanism through network pharmacology and molecular docking. Inhibition kinetics, widely targeted metabolomics, network pharmacology and molecular docking were integrated to systematically elucidate its hypouricemic material basis and molecular mechanism, providing scientific support for resource recycling of this by-product and the manufacture of natural hypouricemic food ingredients.

## 2. Materials and Methods

### 2.1. Materials and Reagents

*Panax notoginseng* stems and leaves (cultivated in Wenshan, Yunnan): dried samples (moisture content: 8.5%, *w*/*w*, determined by drying at 105 °C to constant weight) sealed and stored at 4 °C. Food-grade sodium bicarbonate was purchased from Nanjing Ganzhiyuan Sugar Industry Co., Ltd. (Nanjing, China). Xanthine oxidase (XO, 11.2 U/mg), xanthine (≥99%) and allopurinol (≥98%) were obtained from Shanghai Yuanye Bio-Technology Co., Ltd. (Shanghai, China). PBS buffer was supplied by Biosharp Biotechnology Co., Ltd. (Hefei, China). Methanol, acetonitrile and ammonium acetate were from Merck (Darmstadt, Germany). Formic acid and reference standards were purchased from Shanghai Aladdin Biochemical Technology Co., Ltd. (Shanghai, China).

### 2.2. Extraction Procedure

Exactly 50.0 g of chopped *Panax notoginseng* stems and leaves (dry weight basis, pre-determined moisture content of 8.5%) were subjected to atmospheric steaming treatment at 100 °C for 1 h [21]. After steaming, samples were soaked in food-grade sodium bicarbonate solution (concentration expressed as % *w*/*w* relative to the dry weight of plant material; e.g., 1.0% *w*/*w* indicates 0.5 g NaHCO_3_ per 50 g dried material) [22] at a baseline solid–liquid ratio of 1:15 (25 °C) for 30 min, then supplemented with water to reach the designed solid–liquid ratio. The mixture was boiled and extracted under gentle heating, filtered while hot, and centrifuged at 4000 r/min for 10 min; the supernatant was the aqueous extract. A 10 mL aliquot was vacuum-dried to constant weight at 37 °C to calculate solid yield. The remaining aqueous extract was concentrated under reduced pressure and lyophilized to obtain a dry extract powder. For bioactivity assays, the dried extract was dissolved in PBS buffer to prepare stock solutions at 10 mg/mL (based on dry extract weight), from which working solutions at serial concentrations (0–10 mg/mL) were prepared by dilution. All solutions were stored at 4 °C until analysis.

Solid yield (%) was calculated via Equation (1):

Solid yield (%) = [(M_1_ − M_0_) × V-total/V-sample]/W-raw × 100% (1)

where W-raw is the dry weight of the initial plant material (g); M_0_ is the mass of the empty weighing dish; M_1_ is the total mass after drying; V-total is the total volume of the aqueous extract (mL); V-sample is the volume of the aliquot taken for drying (10 mL).

### 2.3. Single-Factor Experimental Design

The steaming temperature was fixed at 100 °C. The effects of four variables on XO inhibitory rate were investigated individually: steaming time (0–2 h), sodium bicarbonate concentration (0–2%, *w*/*w*, relative to dry material weight), solid–liquid ratio (1:10–1:30, g/mL), and boiling duration (0.5–4 h). Only one factor was altered for each trial, with other parameters kept constant.

### 2.4. Orthogonal Array Design and Factor Level Setting

Four factors (steaming time, sodium bicarbonate concentration, solid–liquid ratio, boiling time), each at three levels, were optimized using an *L*_9_(3^4^) orthogonal array (Table 1). Factor levels: steaming time (0.5, 1, 1.5 h); sodium bicarbonate (0%, 0.5%, 1%); solid–liquid ratio (1:10, 1:15, 1:20 g/mL); boiling time (0.5, 1, 2 h).

The *L*_9_(3^4^) design was selected over response surface methodology because (i) only nine experimental runs are required, (ii) the objective was to identify the optimal factor-level combination rather than fit a predictive model, and (iii) range analysis and ANOVA provide sufficient statistical insight for this purpose.

ANOVA was performed with solid–liquid ratio as the error term (the factor with the smallest range value, R = 3.57). Significance thresholds: α = 0.05 (significant) and α = 0.10 (marginally significant). 

### 2.5. Determination of Xanthine Oxidase Inhibitory Activity and Half-Maximal Inhibitory Concentration (IC_50_)

XO inhibitory activity was determined with minor optimizations based on reported protocols [14,23]. Briefly, 50 μL sample solution was mixed with 50 μL XO solution (0.05 U/mL), preincubated at 37 ± 0.5 °C for 5 min, then reacted with 1.0 mmol/L xanthine solution for 30 min. Absorbance was read at 290 nm to calculate XO inhibition rate. Serial sample concentrations were prepared; GraphPad Prism 9.5.1 was used for four-parameter logistic nonlinear regression to fit dose–response curves and calculate IC_50_ values.

The XO inhibition rate was calculated using Equation (1):

XO inhibition rate (%) = [1 − (D1 − D2)/(D3 − D4)] × 100%

where D_1_ is absorbance of the enzyme-containing sample; D_2_ is the absorbance of the sample without XO (enzyme-free sample); D_3_ is the absorbance of the blank control; D_4_ is the absorbance of the blank control without enzyme.

### 2.6. Inhibition Kinetics Assay

The protocol was slightly modified from published methods [24,25] to identify the reversibility and inhibition mode of *Panax notoginseng* stem and leaf aqueous extract against XO. For reversibility verification: xanthine substrate concentration was fixed at 1.0 mmol/L, extract concentrations ranged 4–7 mg/mL, and reaction rates were measured across XO doses of 0.025–0.15 U/mL. Linear plots of enzyme concentration versus reaction rate determined inhibition reversibility. For inhibition type identification: XO concentration was fixed at 0.05 U/mL, with varied substrate (0.25–1.5 mmol/L) and extract (4–7 mg/mL) concentrations. Lineweaver-Burk double reciprocal plots defined inhibition mode, and Dixon plots yielded the inhibition constant *K*_i_.

### 2.7. UHPLC-MS/MS Metabolite Profiling

#### 2.7.1. Sample Pretreatment

Fifty milligrams of freeze-dried extract powder was blended with 1000 μL ternary solvent (methanol/acetonitrile/water, 1:2:1, *v*/*v*/*v*), followed by 30 s of vortex oscillation. The blended suspension underwent bead milling at 45 Hz over 10 min, then ice-bath sonication lasting 10 min, and a 1 h low-temperature incubation at −20 °C. After 15 min of refrigerated centrifugation (12,000 rpm, 4 °C), 300 μL of clarified upper liquid was passed through a 0.22 μm organic filter membrane and collected into sample vials. Three independent biological replicates were prepared for each experimental group, and each replicate was extracted and analyzed separately. Identical 10 μL portions taken from every single sample were combined to prepare quality control (QC) mixtures used to stabilize instrumental drift throughout detection.

#### 2.7.2. Chromatographic and Mass Spectrometry Conditions

Chromatography: Mobile phase A was water with 0.1% formic acid and 5 mM ammonium acetate; mobile phase B was acetonitrile containing 0.1% formic acid, with identical elution gradients for positive and negative ion modes. Gradient program: 0–1.5 min, 2% B; 1.5–5.0 min, linear ramp to 50% B and held for 4 min; ramp to 98% B within 9.0 min and held for 1 min; 10.0–11.0 min, returned to 2% B, then equilibrated to 14 min. Flow rate: 0.35 mL/min; column temperature: 40 °C; injection volume: 4 μL.

Mass spectrometry: Analysis was performed on an AB6500 QTRAP mass spectrometer (SCIEX, Framingham, MA, USA) equipped with an ESI source, controlled by Analyst 1.6.3 software (SCIEX, Framingham, MA, USA). Ion source temperature: 550 °C; spray voltage: 5500 V (positive), −4500 V (negative); GS1, GS2, CUR: 50, 55, 35 psi; medium collision gas pressure. The instrument was calibrated with polypropylene glycol solution of standard concentration. QQQ detection was operated in MRM mode with segmented ion pair monitoring; DP and CE parameters were optimized.

Metabolite identification followed these criteria: (i) retention time matching with authentic standards where available; (ii) accurate mass error ≤ ±10 ppm; (iii) MS/MS spectral matching against the MetWare MWDB database with reverse and forward matching scores both ≥0.7; (iv) detection in all three biological replicates with relative standard deviation (RSD) of peak area < 30% among replicates; and (v) consistent elution behavior across QC samples with RSD < 15%.

### 2.8. Network Pharmacology

After identification via UHPLC-MS/MS widely targeted metabolomics, the top 30 candidate bioactive metabolites were selected based on the following criteria: (i) relative abundance ranking in the top 10% of all identified metabolites; (ii) belonging to flavonoids, alkaloids, or organic acids, which are classes with documented xanthine oxidase inhibitory or hypouricemic activity in the literature; and (iii) availability of structural information (SMILES or InChI) suitable for target prediction in SwissTargetPrediction. Databases including SwissTargetPrediction, STRING and DAVID were used for target prediction, PPI network construction, and GO/KEGG enrichment analysis with cut-offs: SwissTargetPrediction probability ≥ 0.1; STRING confidence score ≥ 0.4; DAVID *p*-value < 0.05.

### 2.9. Molecular Docking Between Bioactive Constituents of Panax notoginseng Stems and Leaves and Core Targets

Molecular docking was performed using AutoDock Vina (version 1.2.0). The three-dimensional structures of the target proteins-ADRA2A [PDB: 9PLO], ADA [PDB: 3IAR], GAA [PDB: 8CB1], PNP [PDB: 4EAR], and PTGS2 [PDB: 5F19]-were retrieved from the RCSB Protein Data Bank (https://www.rcsb.org/)(accessed on 14 February 2026). Prior to docking, all protein structures were pre-processed in PyMOL (version 2.5.4): water molecules and heteroatoms were removed, polar hydrogens were added, and Gasteiger partial charges were assigned using AutoDockTools (ADT, version 1.5.7). The prepared proteins were saved in PDBQT format. The 2D structures of the candidate bioactive compounds (1-Methyladenosine, Tombozine, Adenosine, and Quercetin 3-Gentiobioside) were retrieved from PubChem (https://pubchem.ncbi.nlm.nih.gov/) (accessed on 14 February 2026) or drawn in ChemDraw (version 20.0) and converted to 3D coordinates. Ligands were energy-minimized using the MMFF94 force field in Open Babel (version 3.1.1), followed by addition of Gasteiger charges, rotatable bond detection, and export to PDBQT format via AutoDockTools. For each target protein, the docking grid box was centered on the active site with dimensions of 40 × 40 × 40 Å (spacing = 1.0 Å). Docking simulations were executed with the following parameters: exhaustiveness = 32, num_modes = 9, energy-range = 4 kcal/mol. AutoDock Vina employs an empirical scoring function combining Lennard-Jones potentials, Coulomb electrostatics, hydrogen bonding, desolvation, and torsional entropy terms to estimate binding free energy (ΔG, kcal/mol). The docking protocol was validated by re-docking co-crystallized ligands (where available) into their respective binding sites. The root-mean-square deviation (RMSD) between the best-scored pose and the crystallographic pose was <2.0 Å, confirming the reliability of the docking parameters. A binding energy ≤ −7.0 kcal/mol was defined as the threshold for strong binding affinity, consistent with established criteria in natural product docking studies. All molecular graphics were rendered in PyMOL or Discovery Studio Visualizer (version 2021).

### 2.10. Statistical Analysis

Raw data were sorted with Excel 2019 (Microsoft Corporation, Redmond, WA, USA). SPSS 25.0 (IBM Corp., Armonk, NY, USA) was used for single-factor and orthogonal test analysis. GraphPad Prism 9.5.1 (GraphPad Software, San Diego, CA, USA) was applied for plotting and fitting IC_50_ plus kinetic parameters.

## 3. Results and Discussion

### 3.1. Single-Factor Results for XO Inhibitory Rate of Panax notoginseng Stem and Leaf Aqueous Extract

**Figure 1:**
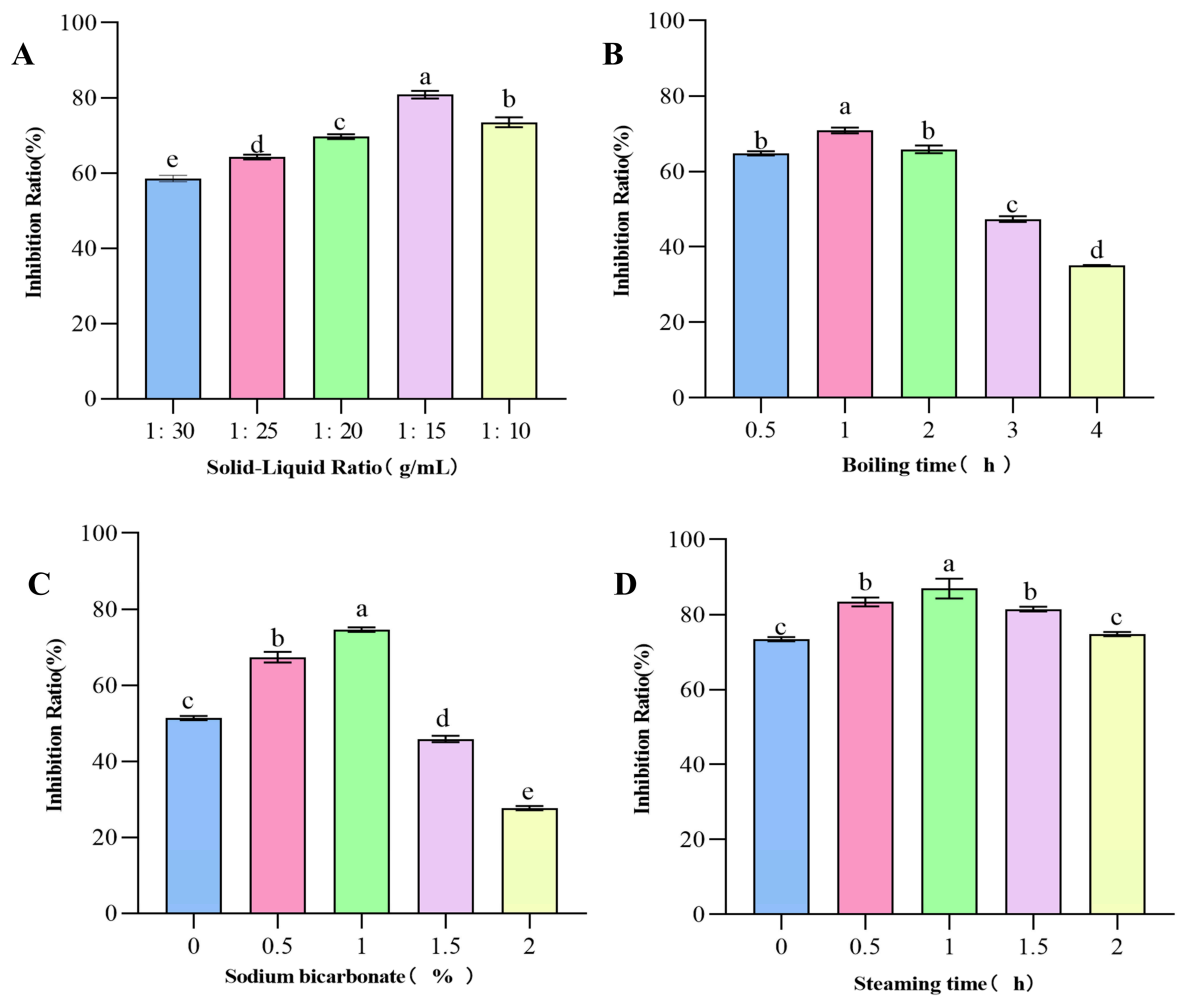
Effects of different extraction parameters on XO inhibitory rate of aqueous extract from *Panax notoginseng* stems and leaves. (**A**) Solid–liquid ratio; (**B**) boiling duration; (**C**) sodium bicarbonate concentration; (**D**) steaming time. Note: Different lowercase letters indicate significant differences between groups (*p* < 0.05).

As shown in Figure 1A, XO inhibitory rate increased significantly when the solid–liquid ratio rose from 1:10 to 1:15, peaking at 82.4 ± 0.9% at 1:15. Moderately increased solvent volume disrupts cell walls and facilitates dissolution of polar bioactive compounds; further increases in the solid–liquid ratio reduced the XO inhibitory rate. This reduction reflects diminished extraction efficiency of bioactive components rather than simple dilution, because the XO inhibitory activity was evaluated at standardized extract concentrations (normalized to dry extract weight) across all samples [26]. Thus, 1:15 was the optimal ratio, and 1:10, 1:15, 1:20 were selected for subsequent orthogonal tests.

Figure 1B reveals that boiling time exerted a rise-then-fall effect on inhibition rate, with the maximum value of 73.1 ± 0.7% at 1 h. Prolonged heating triggers degradation of heat-labile flavonoids and saponins, significantly lowering inhibitory activity [20]. Hence, 0.5 h, 1 h, and 2 h were set as orthogonal levels.

In Figure 1C, XO inhibition rose markedly as sodium bicarbonate concentration increased from 0% to 1%, reaching a maximum of 74.7 ± 0.8%. A mild alkaline medium softens cell walls to boost bioactive leaching, while excessive alkali degrades effective constituents and impairs extraction efficiency [22]. Considering food safety, 0%, 0.5%, and 1% were chosen for orthogonal design.

Figure 1D, exactly 50.0 g chopped *Panax notoginseng* stems and leaves (dry weight basis, moisture content pre-determined as 8.5%) were steamed under atmospheric pressure at 100 °C with gradients of 0, 0.5, 1, 1.5 and 2 h; presents an inverted V-shaped trend for steaming time, where samples steamed for 1 h delivered the highest inhibition (88.3 ± 0.6%). Appropriate steaming breaks cell wall structures to release bioactives, yet prolonged high-temperature treatment causes oxidative degradation and reduced activity [21]. Finally, 0.5 h, 1 h, and 1.5 h were adopted for orthogonal experiments.

### 3.2. Analysis of Orthogonal Experiment Results

**Figure 2:**
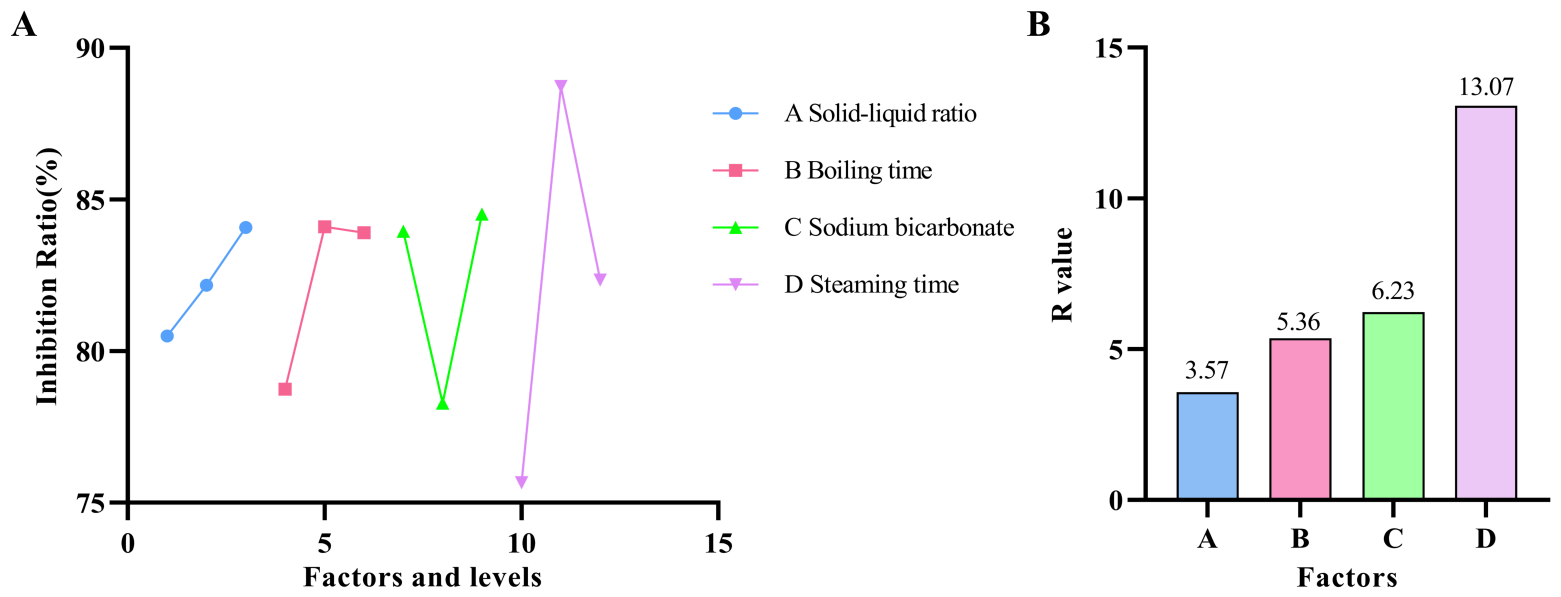
Results of orthogonal experiment on extraction process. (**A**) Effects of four factors on average XOD inhibition rate. (**B**) Range (R) values reflecting the influence intensity of each factor. A, solid–liquid ratio; B, boiling time; C, sodium bicarbonate dosage; D, steaming time.

An *L*_9_(3^4^) orthogonal array was used to optimize extraction parameters, with XO inhibitory rate as the response (Table 2). Range analysis ranked factor influence as: steaming time (R = 13.07) > sodium bicarbonate (R = 6.23) > boiling time (R = 5.36) > solid–liquid ratio (R = 3.57) (Figure 2). The optimal combination was A_3_B_2_C_3_D_2_: solid–liquid ratio 1:20, boiling time 2 h, sodium bicarbonate 1.0%, steaming time 1 h, with a predicted inhibition rate of 92.01%.

ANOVA (Table 3) confirmed the dominance of steaming time (*F* = 13.32, marginally significant at α = 0.10), consistent with range analysis. None of the remaining factors reached significance at either α = 0.05 or α = 0.10. The relatively high critical *F*-value (*F*_0_._05_(2,2) = 19.00) reflects the limited error degrees of freedom inherent in the *L*_9_ design.

Triple verification tests yielded an average inhibition rate of 91.95% (RSD = 0.08%), matching the predicted value and demonstrating reproducibility. The solid yield under optimized conditions (13.50 ± 0.41%) was markedly higher than the control (7.98 ± 0.41%, *p* < 0.05), confirming that combined steaming and sodium bicarbonate treatment enhances extraction efficiency.

Due to the resolution of the *L*_9_(3^4^) orthogonal array, individual two-factor interactions could not be independently estimated from the main effects. The clear dominance of steaming time suggests it is the primary driver of extraction efficiency, with sodium bicarbonate playing a secondary synergistic role. Future work using Box–Behnken or central composite designs would be needed to characterize potential interaction effects.

### 3.3. XO Inhibitory Effect of Panax notoginseng Stem and Leaf Aqueous Extract

#### 3.3.1. In Vitro Inhibitory Activity

**Figure 3:**
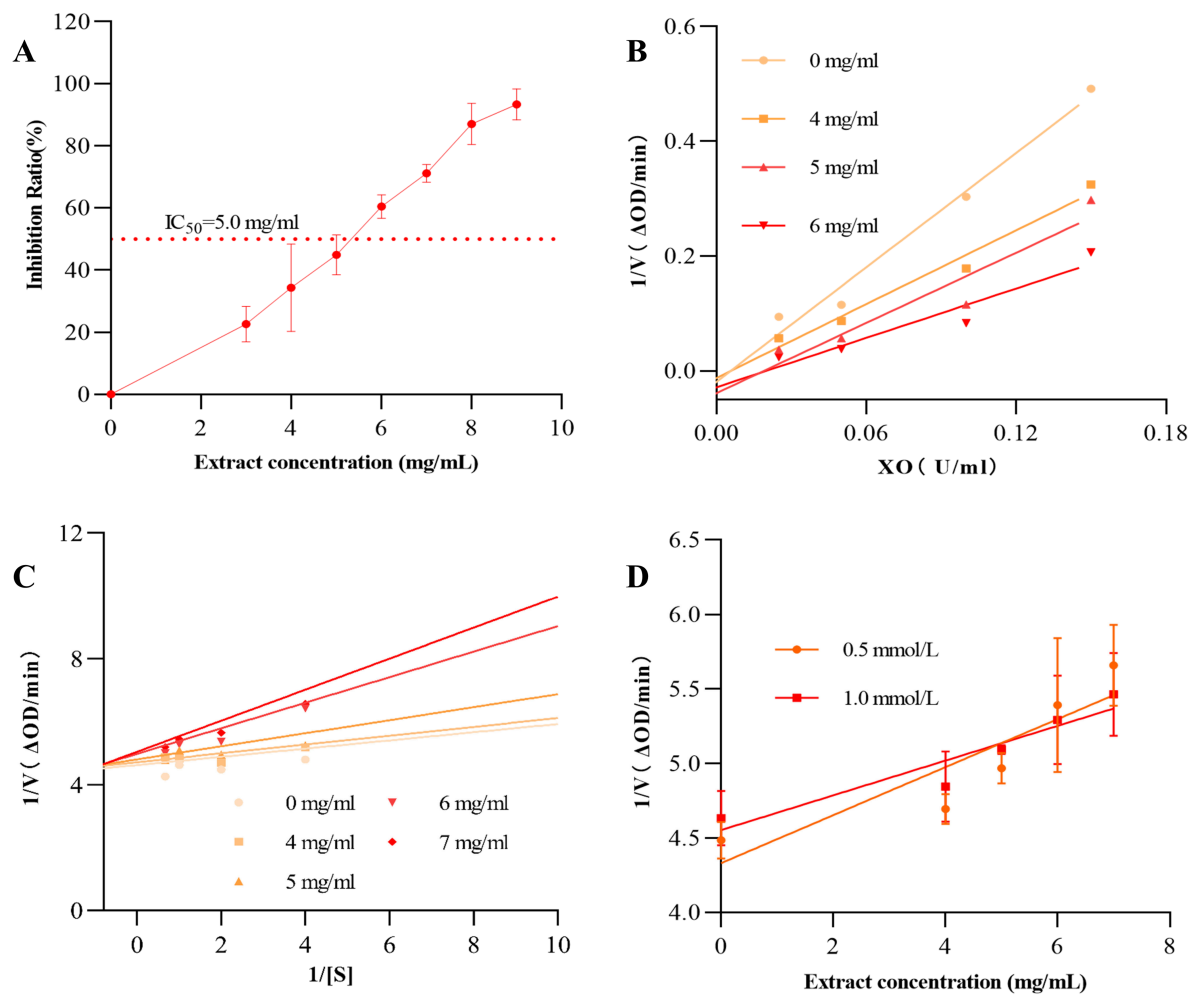
XO inhibitory performance of *Panax notoginseng* stem and leaf aqueous extract. (**A**) Influence of extract concentration on XO inhibitory activity; (**B**) verification of inhibition reversibility; (**C**) identification of inhibition mode; (**D**) determination of inhibition constant *K*_i_. Note: data are presented as mean ± SD (*n* = 3); the dashed line marks the IC_50_ level.

As illustrated in Figure 3A, the XO inhibitory rate of the extract rose significantly with increasing concentration within 0–10 mg/mL. Nonlinear regression fitting gave an IC_50_ value of 5.0 mg/mL. Under identical assay conditions, the positive control allopurinol exhibited an IC_50_ of 46.73 μg/mL. While the extract showed lower potency than the synthetic drug, this is expected for a crude plant extract versus a pure compound. Compared with reported natural XO inhibitors, including *Perilla* Leaf Extract (IC_50_ = 5.8 mg/mL) [27], the *P. notoginseng* stem and leaf extract demonstrated comparable or superior inhibitory activity, supporting its potential as a natural food-grade XO inhibitor.

#### 3.3.2. Inhibition Kinetics Characteristics

All enzyme concentration-reaction rate curves at varied extract concentrations passed through the origin, with slopes declining as extract concentration increased (Figure 3B), confirming reversible inhibition: the extract only suppressed enzyme activity without damaging XO structure [13]. Lineweaver-Burk double-reciprocal plots intersected in the second quadrant (Figure 3C), identifying mixed-type inhibition, where the extract binds both free XO and enzyme-substrate complexes to retard catalytic reactions [14]. All fitted curves had R^2^ > 0.98, indicating a stable, uniform binding mode between the extract and XO [28]. The inhibition constant *K*_i_ = 4.99 mg/mL calculated via Dixon plots reflects strong binding affinity, further supporting the potential of *Panax notoginseng* stem and leaf aqueous extract as a natural XO inhibitor [24,29].

### 3.4. UHPLC-MS/MS Metabolite Analysis

#### 3.4.1. Composition and Classification of Metabolites

**Figure 4:**
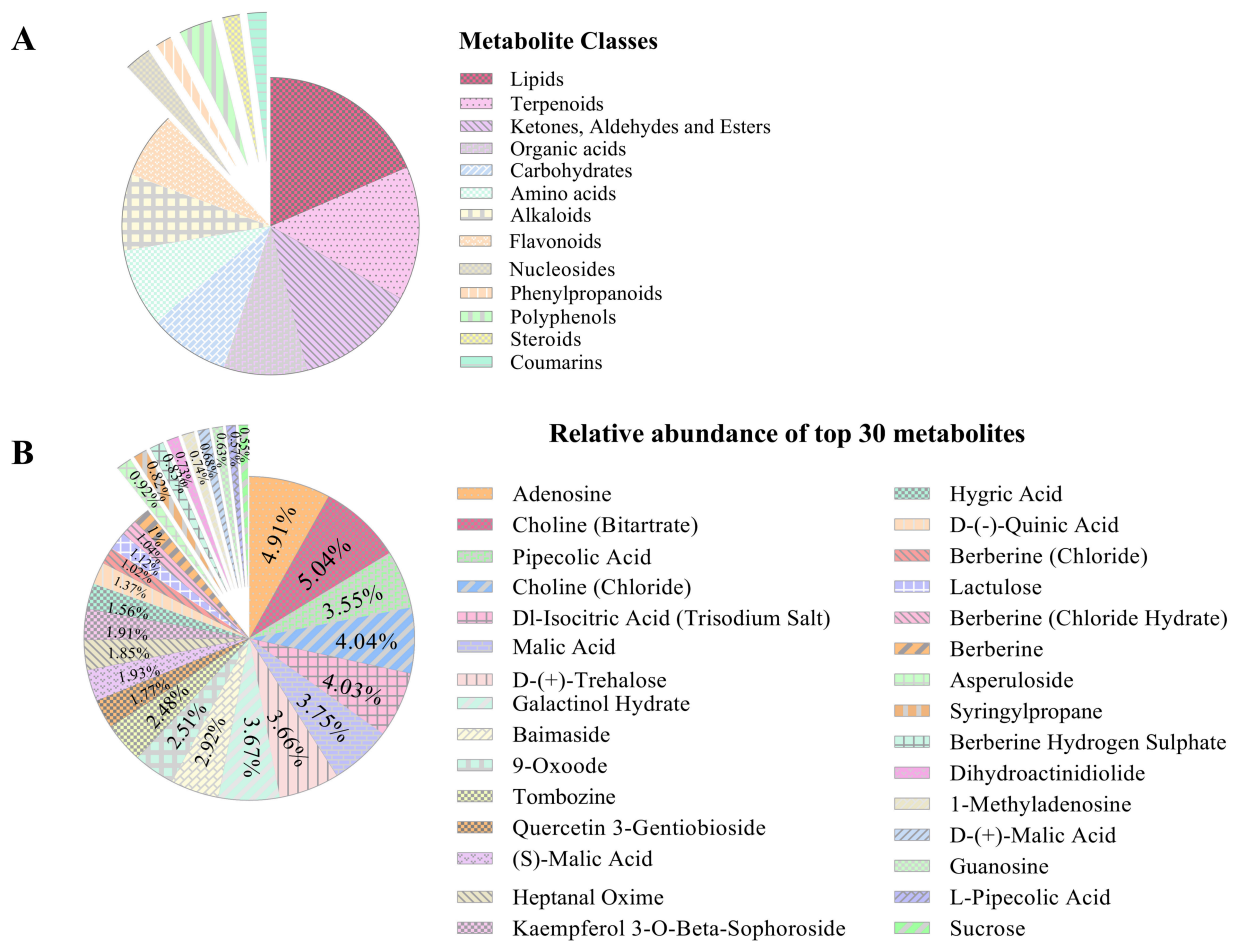
Metabolite profiling of aqueous extract from *Panax notoginseng* stems and leaves. (**A**) Classification distribution of identified metabolites; (**B**) top 30 metabolites ranked by relative abundance. Note: data are presented as mean ± SD (*n* = 3).

Widely targeted UHPLC-MS/MS metabolomics was used to profile components in the optimally extracted aqueous extract of *Panax notoginseng* stems and leaves. In total, 3392 metabolites were identified, including lipids, terpenoids, organic acids, amino acids, alkaloids, flavonoids, saccharides and phenylpropanoids (Figure 4A). Lipids accounted for the largest proportion (15.60%), followed by terpenoids (12.26%), ketones-aldehydes-esters (10.94%), organic acids (7.64%) and flavonoids (6.13%). Flavonoids, alkaloids and organic acids are well-documented constituents with XO-inhibitory, hypouricemic and anti-inflammatory bioactivities [30,31]. The abundant diverse metabolites provide a material foundation for the multi-target biological functions of this extract.

#### 3.4.2. Analysis of High-Abundance Characteristic Constituents

The top 30 metabolites by relative abundance were analyzed (Figure 4B). These metabolites were prioritized based on: (i) highest relative abundance (ranking in the top decile of all identified compounds); (ii) structural classification as flavonoids, alkaloids, or organic acids with documented XO inhibitory or hypouricemic activity; and (iii) availability of structural information for target prediction. Semi-quantitative analysis based on UHPLC-MS/MS peak area intensities revealed that the total relative content of flavonoids and alkaloids accounted for 13.09% of the top 30 metabolites, underscoring their dominant contribution to the bioactive profile. Key bioactive compounds including 1-Methyladenosine [32], Tombozine [33], Quercetin 3-Gentiobioside [34], Adenosine [35] and Guanosine [36] are closely associated with XO inhibition and hypouricemic effects, clarifying the core material basis responsible for the inhibitory activity of *Panax notoginseng* stem and leaf aqueous extract.

### 3.5. Network Pharmacology Analysis

#### 3.5.1. Intersection Analysis of Compound-Disease Targets

**Figure 5:**
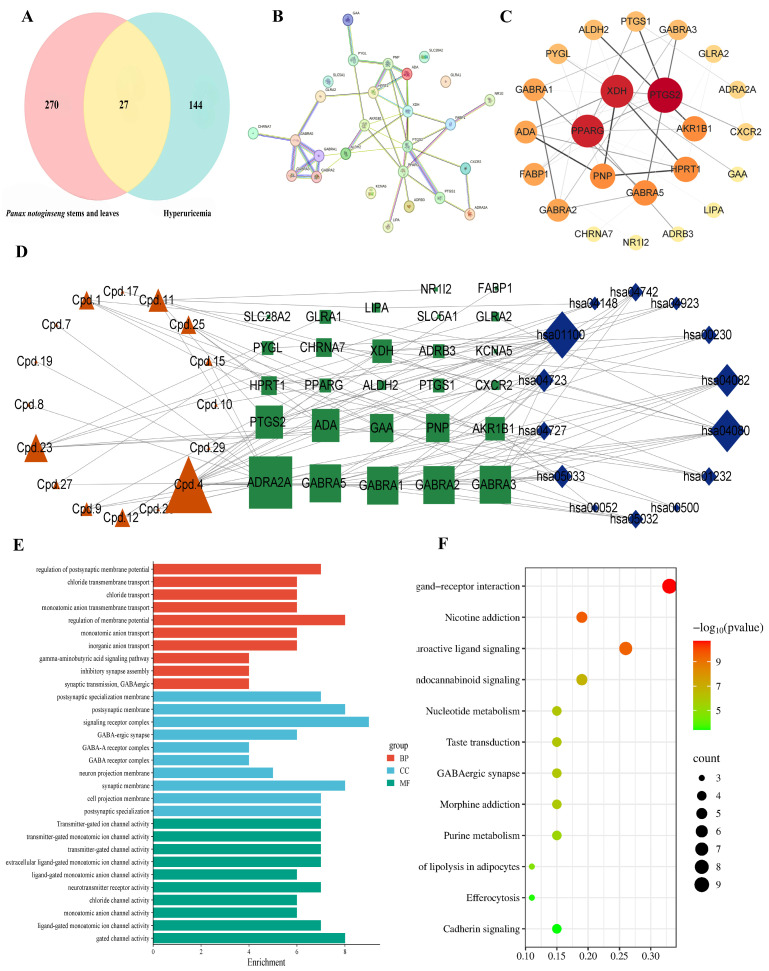
Network pharmacology analysis of *Panax notoginseng* stem and leaf extract against hyperuricemia. (**A**) Overlapping targets between bioactive compounds and hyperuricemia; (**B**,**C**) PPI network of intersecting targets and topological analysis; (**D**,**E**) GO functional enrichment and KEGG pathway enrichment; (**F**) compound-target-pathway regulatory network.

After structural standardization of the top 30 high-abundance metabolites screened by UHPLC-MS/MS, the SwissTargetPrediction database predicted 297 potential human compound targets. Disease targets related to hyperuricemia were retrieved from GeneCards, TTD and DrugBank databases, yielding 171 unique hyperuricemia targets after deduplication. A total of 27 overlapping targets were obtained from the two sets (Figure 5A), revealing that *Panax notoginseng* stems and leaves alleviate hyperuricemia via multi-target pathways.

#### 3.5.2. PPI Protein–Protein Interaction Analysis

The 27 overlapping targets were imported into the STRING database to construct a PPI network. After removing isolated nodes, 23 nodes and 42 interaction edges remained. Topological screening identified core targets: PTGS2 (prostaglandin-endoperoxide synthase 2), XDH (xanthine dehydrogenase), PPARG (peroxisome proliferator-activated receptor gamma), AKR1B1 (aldo-keto reductase family 1 member B1), PNP (purine nucleoside phosphorylase), GABRA5 (gamma-aminobutyric acid type A receptor subunit alpha5), HPRT1 (hypoxanthine phosphoribosyltransferase 1), GABRA2 (gamma-aminobutyric acid type A receptor subunit alpha2), FABP1 (fatty acid-binding protein 1) and ADA (adenosine deaminase). XDH (XO) acts as the critical rate-limiting enzyme for uric acid synthesis [37]; PTGS2 mediates hyperuricemia-triggered inflammatory responses [38]; PPARG regulates renal urate excretion [39]; AKR1B1 and PNP dominate purine metabolism and oxidative stress modulation [40,41], jointly forming the core target network for hypouricemic effects.

#### 3.5.3. GO and KEGG Enrichment Analysis

GO enrichment (*p* < 0.05) of the 27 overlapping targets generated 67 biological process, 20 cellular component and 28 molecular function terms. Enrichment focused on purine nucleotide metabolism, ion transport, inflammation modulation and oxidoreductase activity; target proteins were mainly located on cell membranes and ion channels, demonstrating efficacy through regulating purine metabolism, oxidative stress and inflammation.

Fourteen significantly enriched KEGG pathways were screened, with core pathways including purine metabolism, neuroactive ligand-receptor interaction, GABAergic synapse and general metabolic pathways. Purine metabolism is the central pathway for uric acid production and tightly linked to XDH [42]; neuroactive ligand-receptor interaction modulates urate excretion [43]; GABAergic synapse mitigates hyperuricemia-induced inflammatory and oxidative injury, with synergistic multi-pathway hypouricemic effects [44].

#### 3.5.4. Compound-Target-Pathway Network Analysis

A visual “compound-target-pathway-disease” regulatory network was built, containing 60 nodes and 172 interaction edges. Topological analysis identified key bioactive constituents: 1-Methyladenosine [32], Tombozine [33], Quercetin 3-Gentiobioside [34], Adenosine [35] and Guanosine [36]. These compounds directly interact with multiple molecular targets, which are subsequently enriched in vital pathways associated with hyperuricemia. This reflects a synergistic multi-target, multi-pathway mechanism in which multiple bioactive compounds (multi-component) act in concert on distinct targets, consistent with the network pharmacology paradigm of herbal medicines.

### 3.6. Molecular Docking Verification

**Figure 6:**
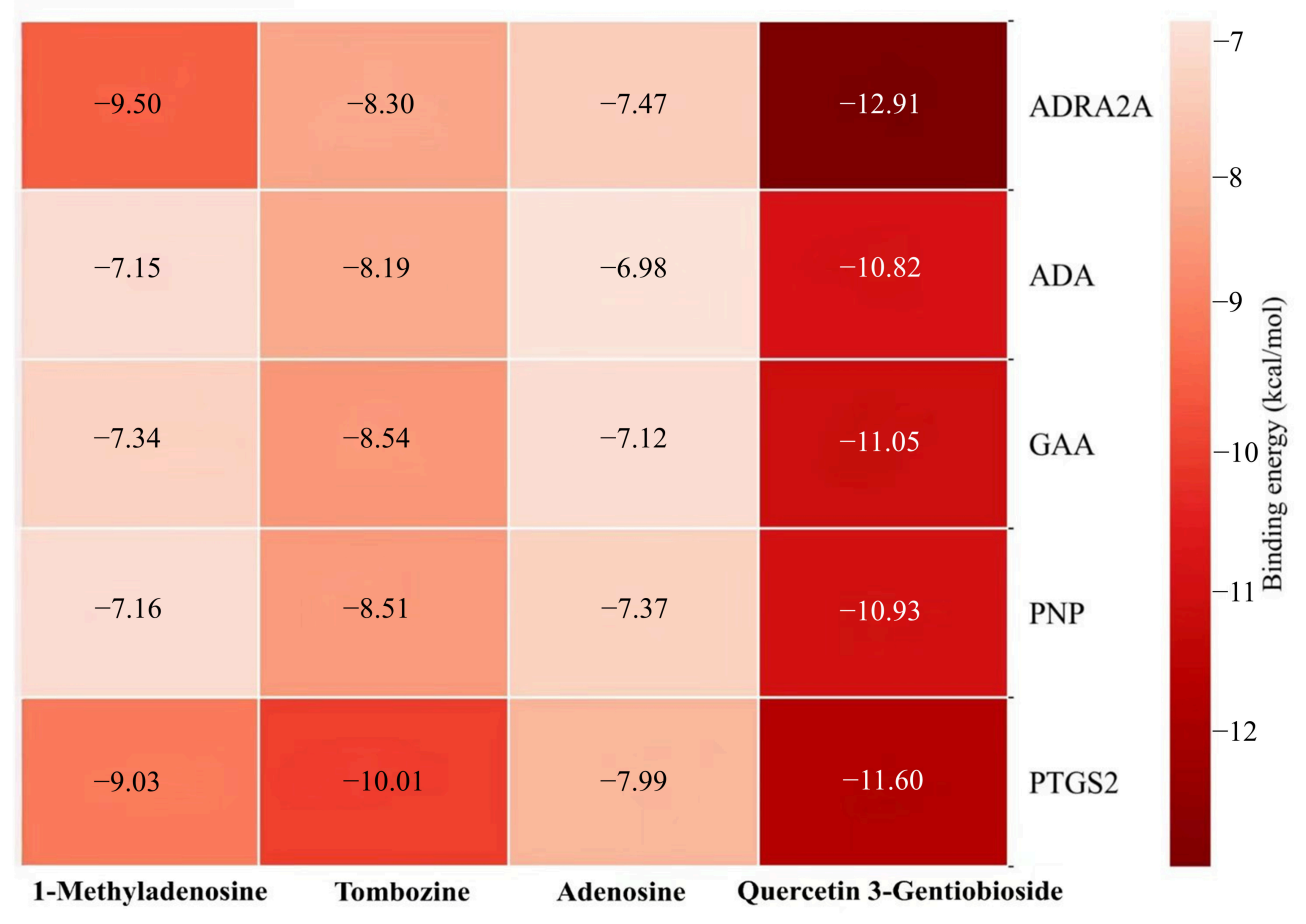
Heatmap of molecular docking binding energies between core bioactive compounds and key protein targets.

**Figure 7:**
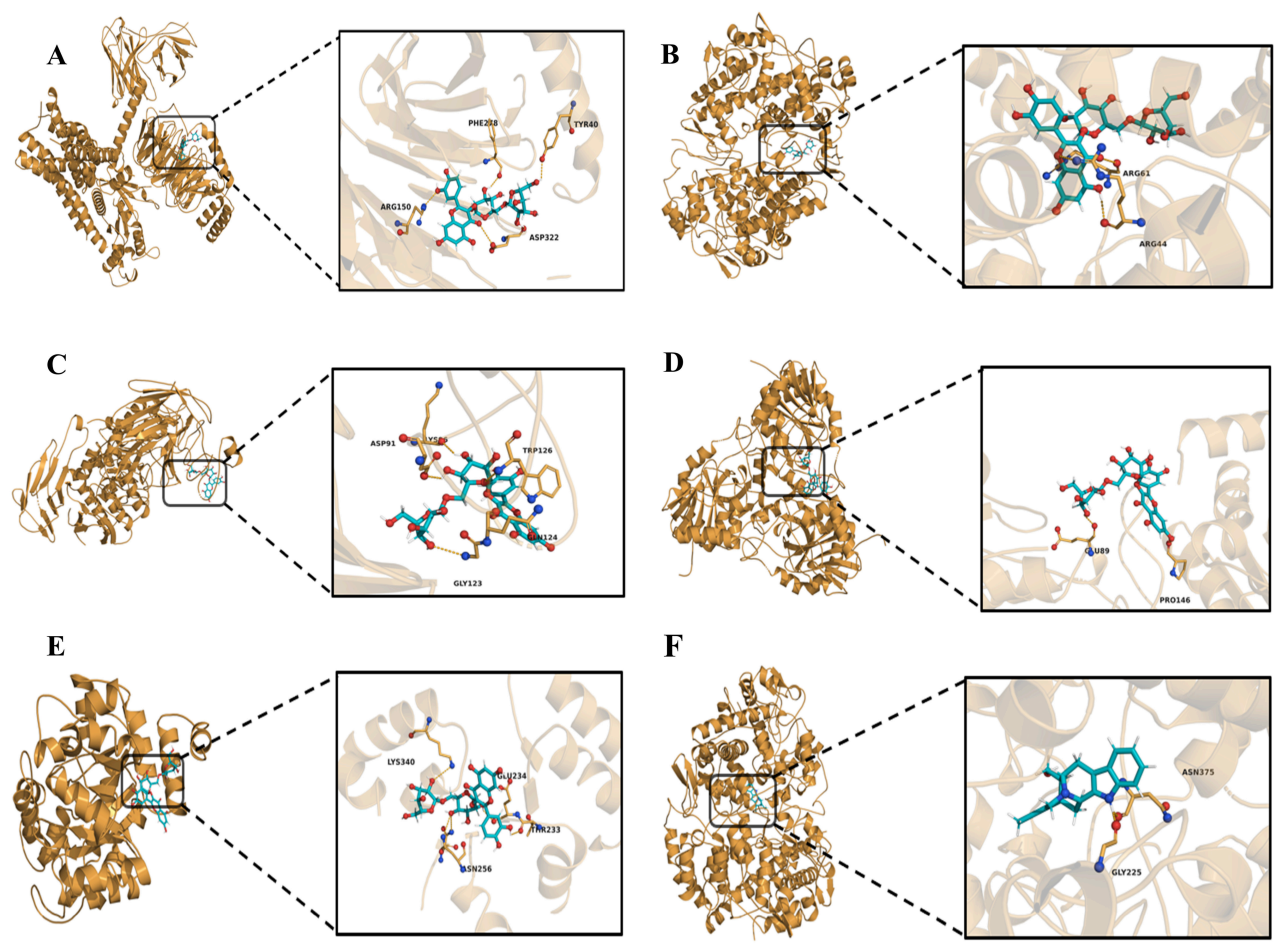
Molecular docking conformations between bioactive constituents and core targets. (**A**–**F**): Docked binding modes of different compound-target pairs. ((**A**) ADRA2A-Quercetin-3-Gentiobioside; (**B**) PTGS2-Quercetin-3-Gentiobioside; (**C**) GAA-Quercetin-3-Gentiobioside; (**D**) PNP-Quercetin-3-Gentiobioside; (**E**) ADA-Quercetin-3-Gentiobioside; (**F**) PTGS2-Tombozine.). Note: Proteins are shown as tan ribbons; ligands are displayed as ball-and-stick models (carbon in cyan, oxygen in red, nitrogen in blue, hydrogen in white); dashed yellow lines indicate hydrogen bonds.

To validate network pharmacology predictions, molecular docking was performed between the top four core bioactive compounds (1-Methyladenosine, Tombozine, Adenosine, and Quercetin 3-Gentiobioside) and the top five key targets (ADRA2A, ADA, GAA, PNP, and PTGS2) (Figure 6 and Figure 7). All compound-target binding energies were lower than −6.5 kcal/mol, with the majority below −7.0 kcal/mol, indicating strong spontaneous binding affinity between *Panax notoginseng* bioactives and core hyperuricemia targets. Quercetin 3-Gentiobioside exhibited the strongest binding, with energies < −10.0 kcal/mol for all targets; its lowest value (−12.91 kcal/mol) occurred with ADRA2A, and it also bound tightly to PTGS2 (−11.60 kcal/mol), GAA (−11.05 kcal/mol), PNP (−10.93 kcal/mol), and ADA (−10.82 kcal/mol). Tombozine [33], 1-Methyladenosine [32] and Adenosine [35] also displayed favorable, stable binding to core targets.

Docking profiles (Figure 7A–F) revealed that Quercetin 3-Gentiobioside embedded within the orthosteric active pockets of ADRA2A, PTGS2, GAA, PNP, and ADA, while Tombozine formed a stable complex with PTGS2, both interacting with critical residues via hydrogen bonds, hydrophobic interactions, and π-π stacking. Quercetin 3-Gentiobioside exhibited the most favorable binding energy toward ADRA2A (−12.91 kcal/mol). As shown in Figure 7A, the compound was deeply embedded into the orthosteric binding pocket of ADRA2A, where it formed hydrogen bonds with residues Lys340, Glu234, Thr233, and Asn256, along with hydrophobic interactions and π-π stacking that collectively stabilized the ligand within the active site, suggesting high-affinity competitive occupancy that may obstruct endogenous ligand access. For PTGS2 (Figure 7B), Quercetin 3-Gentiobioside formed hydrogen bonds with residues Gly225 and Asn375. In the GAA binding pocket (Figure 7C), the ligand established hydrogen bond interactions with Asp91, Trp126, Gln124, and Gly123. Within the PNP active site (Figure 7D), Quercetin 3-Gentiobioside formed hydrogen bonds with Glu89, Pro146, Arg61, and Arg44. For ADA (Figure 7E), Quercetin 3-Gentiobioside formed hydrogen bonds with residues Phe278, Tyr40, Arg150, and Asp322. For PTGS2, Quercetin 3-Gentiobioside (Figure 7B) and Tombozine (Figure 7F) both occupied the same pocket, with Tombozine forming hydrogen bonds with Gly225 and Asn375. Collectively, docking results support a multi-component and multi-target binding pattern of the extract, consistent with stable interactions with pivotal hyperuricemia targets and providing insight into its potential molecular mechanism of XO inhibition and urate reduction [45,46]. It should be noted that network pharmacology and molecular docking are computational predictions based on target databases and docking algorithms; experimental validation, including in vitro enzyme assays and in vivo animal studies, is required to confirm the proposed multi-target hypouricemic mechanism.

## 4. Conclusions

In this work, XO inhibitory activity was used as the indicator to optimize a green steaming-sodium bicarbonate-assisted water extraction process for *Panax notoginseng* stems and leaves. The optimal conditions were steaming for 1 h, 1.0% sodium bicarbonate, boiling extraction for 2 h, and a solid–liquid ratio of 1:20 g/mL. Under these conditions, the aqueous extract delivered an XO inhibition rate of 91.95 ± 0.08% and an IC_50_ of 5.0 mg/mL, with reversible mixed-type inhibition (*K*_i_ = 4.99 mg/mL).

Widely targeted metabolomics detected 3392 metabolites, among which quercetin 3-Gentiobioside and Tombozine were identified as key bioactive constituents. Combined network pharmacology and molecular docking preliminarily suggested that the extract may exert XO inhibitory effects by modulating purine metabolism through core targets including ADRA2A, ADA, GAA, PNP, and PTGS2.

This extraction system uses only pure water and food-grade sodium bicarbonate, offering environmental and economic advantages, low cost, and clean-label compliance. It provides a theoretical basis for the high-value utilization of *P. notoginseng* stem-and-leaf by-products and the development of functional foods with natural XO inhibitory activity. However, this study is limited to in vitro enzymatic assays and in silico predictions. The observed XO inhibition does not necessarily reflect in vivo anti-hyperuricemic efficacy, and the network pharmacology predictions require experimental validation. Therefore, the hypouricemic implications discussed herein should be interpreted as preliminary mechanistic hypotheses rather than established biological efficacy. Future work should focus on in vivo validation using hyperuricemia animal models, pharmacokinetic studies of key bioactive constituents, and long-term safety evaluation.

## Figures and Tables

**Table 1:** Orthogonal experimental design.

No.	Solid–Liquid Ratio (g/mL)	Boiling Time (h)	Sodium Bicarbonate (% *w*/*w*)	Steaming Time (h)
1	1:10	0.5	0	0.5
2	1:10	1.0	0.5	1.0
3	1:10	2.0	1.0	1.5
4	1:15	0.5	0.5	1.5
5	1:15	1.0	1.0	0.5
6	1:15	2.0	0	1.0
7	1:20	0.5	1.0	1.0
8	1:20	1.0	0	1.5
9	1:20	2.0	0.5	0.5

**Table 2:** Results of orthogonal experiments.

No.	A Solid–Liquid Ratio	B Boiling Time	C Sodium Bicarbonate	D Steaming Time	Inhibition Rate %
1	1:10	0.5	0	0.5	72.11
2	1:10	1.0	0.5	1.0	84.86
3	1:10	2.0	1.0	1.5	84.52
4	1:15	0.5	0.5	1.5	74.8
5	1:15	1.0	1.0	0.5	79.7
6	1:15	2.0	0	1.0	92.01
7	1:20	0.5	1.0	1.0	89.31
8	1:20	1.0	0	1.5	87.73
9	1:20	2.0	0.5	0.5	75.18
K_1_	241.49	236.22	251.85	226.99	
K_2_	246.51	252.29	234.84	266.18	
K_3_	252.22	251.71	253.53	247.05	
k_1_	80.50	78.74	83.95	75.66	
k_2_	82.17	84.10	78.28	88.73	
k_3_	84.07	83.90	84.51	82.35	
R	3.57	5.36	6.23	13.07	
Optimal combination	A_3_	B_2_	C_3_	D_2_	

**Table 3:** Analysis of variance (ANOVA).

Source of Variation	Sum of Squares (SS)	Degrees of Freedom (df)	Mean Square (MS)	*F*-Value	Significance
B: Boiling time	55.39	2	27.70	2.88	
C: Sodium bicarbonate	71.28	2	35.64	3.71	
D: Steaming time	256.02	2	128.01	13.32	Marginally significant
Error (*e*)	19.22	2	9.61		
Total	401.91	8			

(Note: Factor A (solid–liquid ratio) was pooled into the error term because it exhibited the smallest effect (R = 3.57), indicating a negligible influence. Critical *F*-values: *F*_0_._05_(2,2) = 19.00, *F*_0_._10_(2,2) = 9.00. Factor D showed a marginally significant difference at α = 0.10.).

## Data Availability

The original contributions presented in this study are included in the article. Further inquiries can be directed to the corresponding authors.
